# Solution of Gall Stones

**Published:** 1890-05-03

**Authors:** 


### THE PRACTITIONER'S MIRROR AND RETROSPECT.

*[The Editor will be glad to receive offers of co-operation and contributions from members of the profession. There is no doubt that much valuable material full of interest and instruction is at present lost to science. This is largely so in the case of (1) the younger and less-known members of the hospital staff (2) those connected with cottage hospitals, and (3) the great body of medical practitioners not attached to any institution. The co-operation of all is cordially invited to enable the Editor to make* THE HOSPITAL *of the utmost possible use and value to the profession. Specialists willing to review books on their special subjects are invited to communicate this fact at once. All letters should be addressed to* THE EDITOR, THE LODGE, PORCHESTER SQUARE, LONDON, W.]

### MEDICINE.

### SOLUTION OF GALL STONES.

The solution of gall stones by agents introduced into the circulation has long been a desideratum. Durande's prescription of ether and turpentine is now generally discredited, and the treatment by large quantities of alkaline laxative waters, as those of Carlsbad, though effective in the case of small stones and as a preventive measure, is by no means so successful with large concretions. Some time since we noticed the American treatment with huge doses of olive oil, which appeared to give very satisfactory results, though in itself somewhat repugnant to most persons. Dr. Stiller, of Buda-Pesth, now calls attention to the value of salicylate of soda, which he was induced to try in consequence of its having been recommended by English physicians somewhat vaguely for diseases of the liver. He states, as the result of his observations, that he finds "no more certain and speedy remedy for gall stones" than the salicylate in ½ gram. (8 grain) doses four times daily in half a glass of soda water or effervescing lemonade, to which he usually adds about ⅙ grain of extract of belladonna. The attacks of colic soon become fewer and less severe, so that morphia injections are rarely necessary after the first few days, and before many weeks have elapsed he sends his patients to Carlsbad or some similar springs. The diet, though nourishing, should be as fluid as possible.

